# Mitochondrial genomes of the key zooplankton copepods Arctic *Calanus glacialis* and North Atlantic *Calanus finmarchicus* with the longest crustacean non-coding regions

**DOI:** 10.1038/s41598-017-13807-0

**Published:** 2017-10-20

**Authors:** Agata Weydmann, Aleksandra Przyłucka, Marek Lubośny, Katarzyna S. Walczyńska, Ester A. Serrão, Gareth A. Pearson, Artur Burzyński

**Affiliations:** 1 Institute of Oceanology, Polish Academy of Sciences, Sopot, 81-712 Poland; 2 University of Gdansk, Institute of Oceanography, Gdynia, 81-378 Poland; 3 University of Algarve, CCMAR, CIMAR, Faro, 8005-139 Portugal

## Abstract

We determined the nearly complete mitochondrial genomes of the Arctic *Calanus glacialis* and its North Atlantic sibling *Calanus finmarchicus*, which are key zooplankton components in marine ecosystems. The sequenced part of *C*. g*lacialis* mitogenome is 27,342 bp long and consists of two contigs, while for *C. finmarchicus* it is 29,462 bp and six contigs, what makes them the longest reported copepod mitogenomes. The typical set of metazoan mitochondrial genes is present in these mitogenomes, although the non-coding regions (NCRs) are unusually long and complex. The mitogenomes of the closest species *C. glacialis* and *C. finmarchicus*, followed by the North Pacific *C. sinicus*, are structurally similar and differ from the much more typical of deep-water, Arctic *C. hyperboreus*. This evolutionary trend for the expansion of NCRs within the *Calanus* mitogenomes increases mitochondrial DNA density, what resulted in its similar density to the nuclear genome. Given large differences in the length and structure of *C. glacialis* and *C. finmarchicus* mitogenomes, we conclude that the species are genetically distinct and thus cannot hybridize. The molecular resources presented here: the mitogenomic and rDNA sequences, and the database of repetitive elements should facilitate the development of genetic markers suitable in pursuing evolutionary research in copepods.

## Introduction

Copepods of the genus *Calanus* are widely spread in the world ocean and are particularly important for the ecosystems of polar and sub-polar regions. In the northern hemisphere, the distributions of each species coincide with latitudinal gradients. In the northern North Atlantic Ocean there is *C. finmarchicus* with its distribution centres in the Labrador and Norwegian Seas. Further north there are *C. glacialis*, an Arctic shelf water species, and *C. hyperboreus*, a deep oceanic one^1,2^. Most zooplankton biomass in the Arctic shelf seas is formed by the grazer *C. glacialis* ^3–5^, which, in this lipid-based food web, is a key link between low-energy microalgae and higher trophic levels, including zooplankton, fish and planktivorous seabirds^6–8^. The species are usually sympatric, especially at the limits of their distribution ranges. However, with the recently observed climatic changes, copepods are shifting their spatial distribution northwards, mostly due to seawater temperature rise^9–11^.

According to Bergmann clines, body sizes of ectotherm species within a genus, as well as of populations within a species, increase along temperature gradients towards colder areas^12^. Such a relationship has also been reported for copepods of the genus *Calanus*, that generally live longer and have larger body sizes in higher latitudes of the Northern hemisphere^2^. The functional links between body and genome size in copepods, and their physiological, morphological and ecological consequences have been studied by different authors^13–15^ leading to conclusions that genome size is a key trait of organisms, although its adaptiveness is still unclear.

*Calanus* copepods are influenced by ongoing climate changes, especially the increasing temperatures in the Arctic and sub-Arctic waters and sea-ice loss. Increasing numbers of *C. finmarchicus* are transported into the Arctic with the West Spitsbergen Current, which is the continuation of the North Atlantic Current, carrying boreal species northwards^11^. At the same time, due to the sea temperature rise, changes in population structure are observed, possibly leading to earlier spawning and accelerated development, which in turn could lead to food web reorganization in the sub-Arctic regions^16^. Its sibling species, *C. glacialis*, which needs energy from the ice algal bloom to fuel its reproduction, is also affected by increasing temperatures^17^ and the loss of sea ice. This may lead to a mismatch between the primary production peaks and the reproductive cycle of this key species, with negative consequences for the higher trophic levels^4^. Due to the important role of both *Calanus* species in the lipid-driven Arctic and sub-Arctic food webs, major functional changes in these marine ecosystems are anticipated under climate warming.

In spite of the importance of the calanoid copepods for the ecosystems and as indicators of ongoing climatic changes, there is still a lack of consensus about the relationships among their lineages, in which high latitude species are especially under-represented. Mitochondrial DNA is useful for evolutionary studies and for reconstructing the phylogenetic relationships of copepods, which is challenging due to the morphological complexity of their taxonomic features^18,19^. Few copepods have their mitochondrial genomes published so far^20^, of which only one is an Arctic calanoid, *Calanus hyperboreus* ^21^. In this genus, within the superorder Gymnoplea^22^, only one species of the northwest Pacific, *Calanus sinicus*, has its mitochondrial genome also known^23^. Both *Calanus* genomes exhibit some unusual features, including the longest control region reported for a crustacean, a large tRNA gene cluster, and reversed GC (guanine-cytosine content) skews in most of the protein-coding genes in *C. hyperboreus* ^21^, and the concurrence of multiple non-coding regions and a reshuffled gene arrangement in *C. sinicus* ^23^. The known mitogenomes of Gymnoplea, having the length of 17,910 bp in the first one and 20,460 bp in the latter species, are also considerably longer than other known copepod mitochondrial genomes, all in the superorder Podoplea. Due to these unusual characters, the conservation of mitochondrial gene order in copepods has been questioned by some authors^23,24^, while others claim that copepod mitochondrial genomes retained pan-crustacean features and conserved calanoid-specific patterns^21^ or adapted to harsh environments by mitogenome rearrangements^24,25^.

We aimed to sequence the mitochondrial genomes of the Arctic *Calanus glacialis* and its sibling species of the North Atlantic, *Calanus finmarchicus*, to study morphological and evolutionary relationships between high latitude copepods, and to obtain a database which will facilitate the development of genetic markers suitable in pursuing evolutionary processes in *Calanus* copepods, which in the future could be linked with climate change in the Arctic and sub-Arctic regions.

## Results

### Structure and composition of *C. glacialis* and *C. finmarchicus* mitogenomes

**Figure 1:**
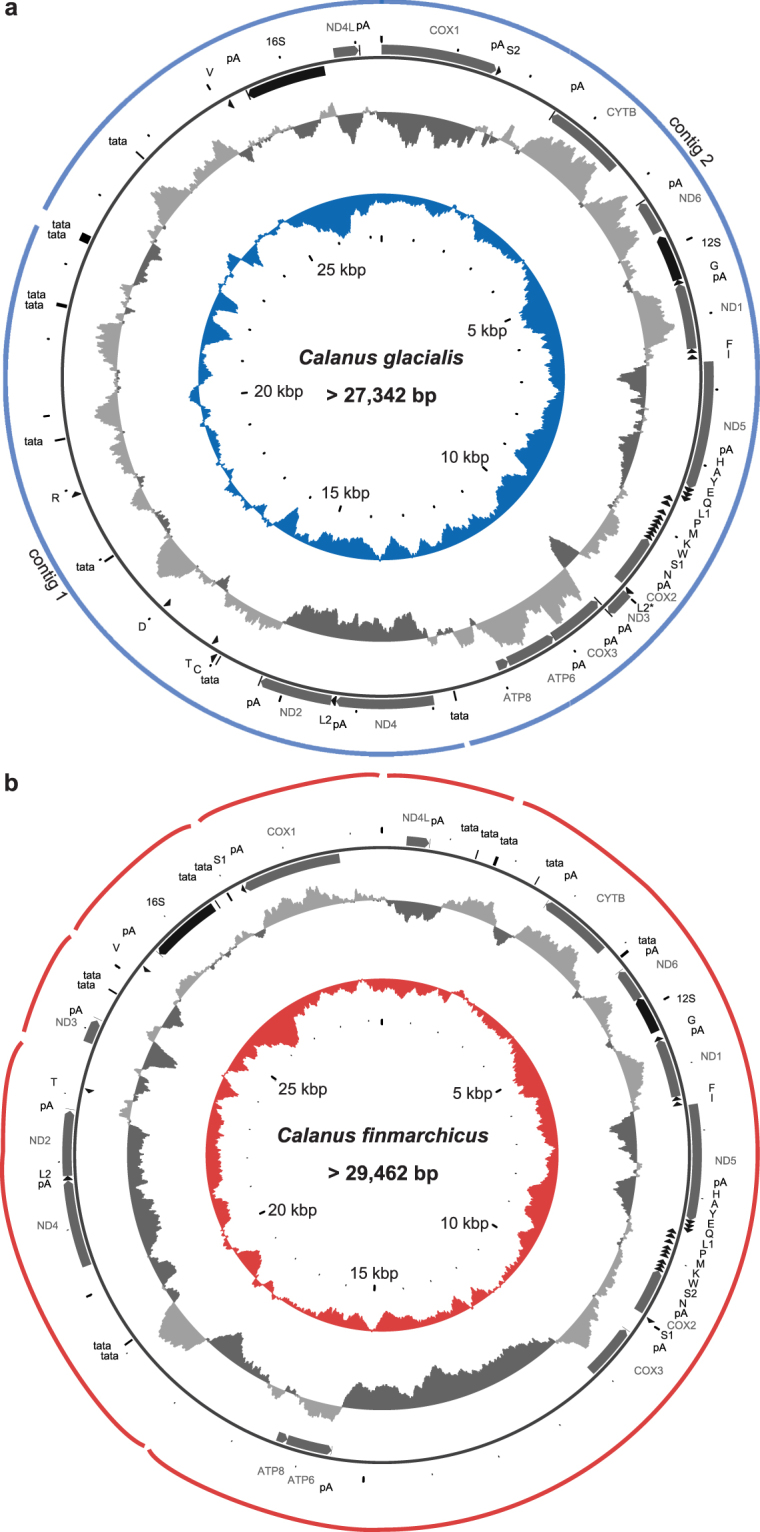
Mitochondrial genomes organization: (**a**) of the Arctic copepod *Calanus glacialis* and (**b**) of the North Atlantic *Calanus finmarchicus*. The span of the assembled contigs is indicated by the outer arches. All protein coding genes (CDS) are labelled by the names of the encoded proteins, two *rrna* genes are labelled 16 S and 12 S for large and small subunit, respectively. The *trn* genes are labelled by the one letter code of the respective amino acid, distinguishing the two possible anticodons by a number, wherever applicable. The supranumerary *trnL* ^*TAA*^ gene in (**a**) is distinguished by an asterisk. Direction of transcription is indicated by the position and direction of the arrows, with clockwise transcribed genes on the outside and anticlockwise transcribed genes on the inside of the circle representing the genome. Positions of polyadenlylation sites (pA) and microsatellite-like repeats (tata) are also indicated. The two inner circles represent local compositional bias, calculated in a 300 bp long window. The light grey parts of the first circle represent positive AT skew bias while the dark grey parts of this circle represent negative AT skews. The inner, color circle represents local GC (guanine-cytosine) content, relative to the reference 50%.

For *C*. g*lacialis*, both NGS assemblers produced similar assembly. Despite a good coverage (approx. 100 fold), they were unable to retrieve the complete mitochondrial genome in one contig from the genomic data set. Instead, two contigs of 9,686 bp and 17,656 bp were generated. Based on the mapping of PE reads and the similarity of repeats at respective contig ends, the two contigs were provisionally joined to form a circular entity. The resulting genetic map of *C. glacialis* mitochondrial genome is presented in Fig. 1a. Numerous attempts to bridge the remaining gaps by Long Range PCR consistently failed. The sequenced part of the mitogenome is 27,342 bp long, but the complete genome could potentially be much longer.

For *C. finmarchicus* the assembly was even more fragmented and consisted of six relatively long contigs, with the following lengths: 1) 1,431 bp, 2) 17,682 bp, 3) 4,129 bp, 4) 1,417 bp, 5) 2,074 bp, and 6) 2,729 bp. Each of the contigs contained appreciable amount of the non-coding regions (NCRs). It was possible to scaffold the contigs only provisionally (Fig. 1b), despite PCR-based and *in silico* attempts. Nevertheless, the sequenced part of the mitogenome (29,462 bp) suggests that this mitogenome is even larger than that of *C. glacialis*.

The typical set of mitochondrial genes is present in these mitogenomes but NCRs are unusually long and complex. Surprisingly, neither genome is AT-rich, with the overall AT content of the sequenced part at 58.3%, the same in both species. Moreover, the strand asymmetry, typically associated with mitochondrial DNA, is negligible: the difference in molecular weight between the two DNA strands is 0.05%, the G + T content of the “heavy” strand is 51.7% only, in *C. glacialis*.

**Figure 2:**
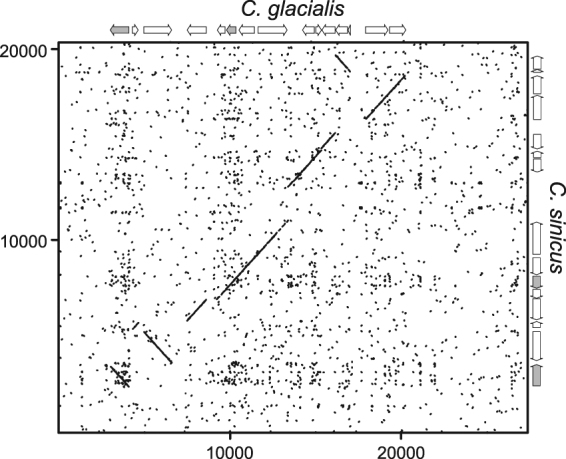
Dotplot comparison of the two *Calanus* mitogenomes: *C. glacialis* and *C. sinicus*. Forward and reverse dotplots were overlaid to show the cases of reversed fragments. Schematic maps of both mitogenomes are on the margins, with protein coding genes (CDS) in white and *rrn* genes in grey. It is evident that non-coding parts of the genomes lack homology comparable with the homology of coding parts. The genes are also remarkably similar in length.

**Figure 3:**
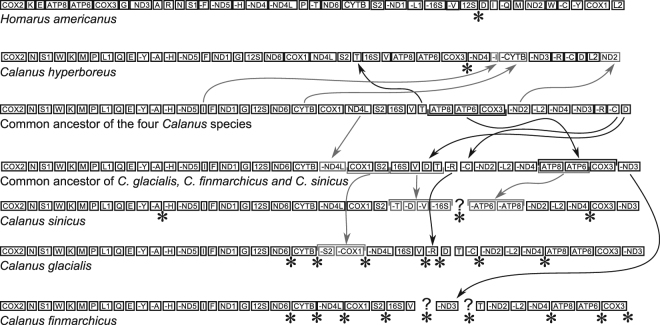
Parsimonious evolutionary scenario leading to the four extant gene orders. CREx analysis implies that *C. sinicus* and *C. glacialis* gene orders can be derived from each other by as little as three reversed translocations. The gene order represented by *C. hyperboreus* is substantially more distant, separated by more than seven events. The ancestral gene orders proposed here fit these data well, with two events separating *C. sinicus* and *C. glacialis* mitogenomes from their common ancestor and the further four events separating common ancestor of all three species. Simple translocations are represented by black arrows, translocations with reversals by grey arrows. Black asterisks show the positions of major non-coding regions. Note that the non-sequenced part of *C. sinicus* and *C. finmarchicus* genomes (indicated by the question marks) will most likely contain the missing *trn* genes (C, D and R), which also fits this scenario well.

The comparison of the overall structures of the mitogenomes of *C. glacialis* and *C. sinicus* by dotplot revealed a substantial similarity in coding parts and lack of any homology outside coding regions (Fig. 2). There were also similarities in gene order, with a long stretch of genes shared between *C. glacialis* and *C. sinicus*. To formally analyse the relationship between gene orders of the four available mitogenomes of *Calanus* species, and calculate the rearrangement distances, CREx analysis was performed (Fig. 3). It confirmed greater rearrangement distance between *C. hyperboreus* and the pair of *C. sinicus* and *C. glacialis*, the latter two consistently presenting a closer relationship. The comparisons involving *C. finmarchicus* were limited to contig 2 and indicated one additional translocation involving *nd3* gene, otherwise this mitogenome may have the same gene order as the MRCA of *C. glacialis* and *C. sinicus*.

### Non-coding regions

There are at least three NCRs longer than 300 bp within the 17 kbp contig and only 2.5 kb of the 10 kbp contig is coding in *C. glacialis*. In addition, the ends of both contigs contain NCRs characterized by complex repeats, hence their length is uncertain. Interestingly the NCRs are not particularly A + T-rich (Fig. 1). They also do not contain any orphan open reading frames of a significant length. The situation in *C. finmarchicus* mitogenome is similar, only more tandem arrays of TATA repeats are present than in *C. glacialis*. There is no sequence similarity between NCRs of the two species, despite their similar composition.

### Ribosomal RNA and tRNA genes

Surprisingly, the most A + T rich parts of the mitogenomes are the two *rrn* genes (Fig. 1). They are relatively short (1125 and 656 bp in both mitogenomes), but do align well with the respective genes of *C. sinicus*. The overall nucleotide p-distance between *C. glacialis* and *C. sinicus 16 S* genes is 20%, for *12 S* gene the distance is 14%. For the pair of mitogenomes presented here these distances are 14% and 13%, respectively.

The 22 expected *trn* genes are present in the *C. glacialis* mitogenome. They form a set typical for the invertebrate mitochondrial genetic code. All four-fold degenerated codons are served by a tRNA with T at the first anticodon (wobble) position within *trn* genes. The two-fold degenerated families have either T or G at this position, with the single exception of the *trnM* with C at the wobble position. Surprisingly, two copies of *trnL2*, both with TAA anticodon were found. They both should form typical, stable cloverleaf structures according to *arwen*, with comparable bitscores (30 and 31). One of the genes was located between *nd2* and *nd4*, in the first contig, while the alternative version was present between *cox2* and *nd3* in the second contig (L2* in Fig. 1). Since there is no way to dismiss one of them as a non-functional pseudogene, it must be tentatively assumed that there are 23 *trn* genes in the *C. glacialis* mitogenome. Twelve of the *trn* genes are found in one big cluster between *cox2* and *nd5*.

The sequenced part of *C. finmarchicus* mitogenome does not contain all expected *trn* genes, the *trnC, trnR*, and *trnD* are missing. Notably, according to the analysis of gene order evolution, they are expected to be within the missing parts of the mitogenome (Fig. 3). One supranumerary *trn* gene was found in *C. finmarchicus*: the second copy of *trnS1* gene, located in exactly the same space where the supranumerary *trnL2* was found in *C. glacialis* (Fig. 1).

### Protein-coding genes

All protein coding genes (CDS) typically expected in animal mtDNA are present in the *C. glacialis* and *C. finmarchicus* mitogenomes. Given the unusually loose overall structure of these genomes, it is surprising to note that the CDS lengths are largely unaffected, with the overall number of encoded amino acids at 3698-3701, within two amino acid difference of *C. sinicus* (Table 1). There are also seven new cases of incomplete stop codons, in each of the cases the completion of the stop codon by polyadenylation of the transcript was confirmed by transcript mapping. These incomplete stop codons end *cytb*, *nd1*, and *nd5* genes in both species and *atp6* in *C. finmarchicus*.

**Table 1:** Lengths of all mitochondrial protein coding genes (CDS) of four *Calanus* species (in base pairs, bp) and the lengths of the corresponding amino acid chains (aa), with their relative standard deviation (RSD).

CDS	*C. glacialis*		*C. finmarchicus*		*C. hyperboreus*		*C. sinicus*		RSD
name	bp	aa	bp	aa	bp	aa	bp	aa	%
*atp6*	714	237	712	**237**	708	235	711	236	0.41
*atp8*	162	53	162	53	159	52	162	53	0.95
*cox1*	1557	518	1557	518	1546	**515**	1548	515	0.34
*cox2*	705	234	705	234	704	**234**	705	234	0.00
*cox3*	792	263	792	263	792	263	792	263	0.00
*cytb*	1133	**377**	1132	**377**	1137	378	1137	378	0.15
*nd1*	919	**306**	919	**306**	915	304	917	**305**	0.31
*nd2*	969	322	969	322	969	322	969	322	0.00
*nd3*	354	117	354	117	354	117	354	117	0.00
*nd4*	1302	433	1302	433	1302	433	1302	433	0.00
*nd4l*	336	111	336	111	336	111	336	111	0.00
*nd5*	1711	**570**	1712	**570**	1710	569	1723	**574**	0.39
*nd6*	474	157	483	160	474	157	480	159	0.95
All CDS		3698		3701		3690		3700	0.14

The lengths of amino acid chains are obtained by dividing the length of nucleotide sequence by three and subtracting one (for the stop codon), except for the eleven cases of genes with incomplete stop codons (in boldface).

**Figure 4:**
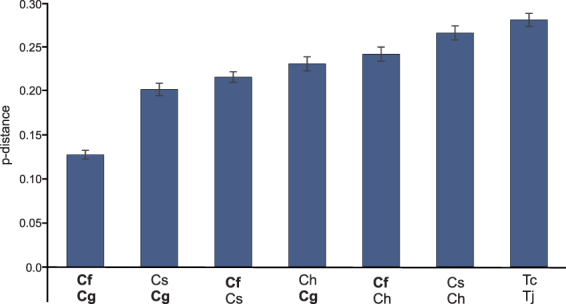
Estimates of intragenic polymorphism in *Calanus* and *Tigriopus*. Four *Calanus* (*C. finmarchicus, C. glacialis*, *C. sinicus*, and *C. hyperboreus* labelled as Cf, Cg, Cs and Ch, respectively) and two *Tigriopus* (*T. japonicus* and *T. californicus*, Tj and Tc) species were compared. The dataset comprised concatenated amino acid alignments of all mitogenome CDS. All positions with gaps and missing data were eliminated. There were a total of 3306 positions in the final dataset. The number of amino acid differences per site (p-distances) between sequences, with standard error estimates are shown. The average, uncorrected amino acid p-distance between *Calanus* and *Tigriopus* genera is slightly higher than 0.5.

Genetic distances between the congeneric species were calculated based on concatenated CDS alignments (Fig. 4). The closest relative of *C. glacialis* is *C. finmarchicus*, followed by *C. sinicus*, however the overall genetic distance between them is still substantial. Uncorrected amino acid p-distances are larger than 12% in all cases. The distance between the two *Tigriopus* species, shown for comparison, is nevertheless even higher.

**Figure 5:**
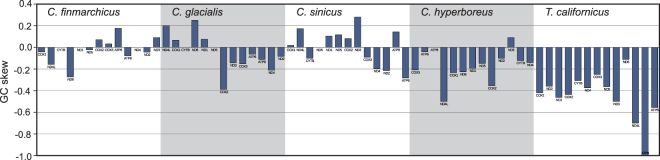
Compositional bias at four-fold degenerate sites in CDS of the four *Calanus* species and *T. californicus*. GC (guanine-cytosine) skew was calculated separately for each gene, based only on the most neutral sites, that is four-fold degenerated positions. This index is particularly sensitive to mutational pressure associated with the relative position of the gene and the origin of replication. Native directions and relative positions of the genes were used, the charts start at the position of a major NCR in each species.

It is important to consider the local compositional biases in the highly rearranged and variable structure of the *Calanus* mitogenomes. Local AT skew (Fig. 1) shows excess of T over A in all CDS transcripts. However, subtle mutational pressures can only be traced by calculating skews at four-fold degenerated (selectively neutral) sites, as these should not be affected by compositional biases of encoded proteins. Particularly the GC skew at neutral sites is considered indicative of the orientation and proximity of regulatory elements. In the studied *Calanus* mitogenomes, the GC skew at neutral sites is highly irregular: the values are small and change both within each genome and between the congeneric genomes for the same genes (Fig. 5).

### Nuclear background

Nuclear genomes of the two *Calanus* species are expected to be complex and sequencing them will require much more effort than a single NGS run. Nevertheless, the sequencing gave us the opportunity to check some basic properties of *C. glacialis* and *C. finmarchicus* nuclear genomes.

**Figure 6:**
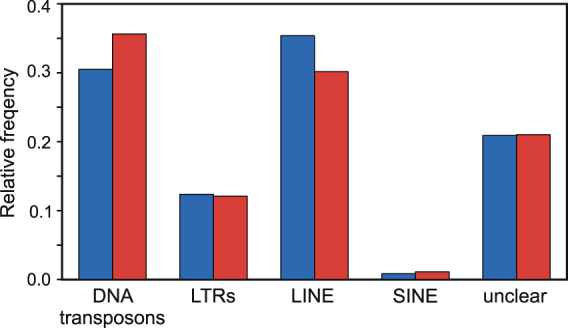
The classification of repetitive elements from the nuclear genome of *C. glacialis* (blue) and *C. finmarchicus* (red). The elements represented by k-mer coverage greater than 45 were assembled by *tedna*, resulting in 4582 elements from *C. glacialis* and 4204 elements from *C. finmarchicus*. These elements were then roughly classified by *Teclass*.

In principle, it should be possible to estimate the size of the haploid genome from the distribution of unique k-mers^26^, providing the sequencing coverage is adequate. With 4.5 × 10^10^ bp obtained in the present study, the k-mer distribution analysis should yield a meaningful result if the sampled genome was similar to the human genome in size. The distributions of k-mer frequencies in our data did not have non-trivial maxima (data not shown), indicating large genomes, rich in repetitive sequences. An attempt has been made to assembly the most repetitive parts of the nuclear genomes. The two species are remarkably similar, but *C. glacialis* has more repetitive elements. Both the number of identified elements was higher: 4582 *vs*. 4204 for *C. finmarchicus* (at the same arbitrary assembly threshold of 45 × ), and they represented bigger fraction of the genome (30% *vs* 25%). The expansion of LINE elements in the genome of *C. glacialis* is probably responsible for this difference (Fig. 6).

**Figure 7:**
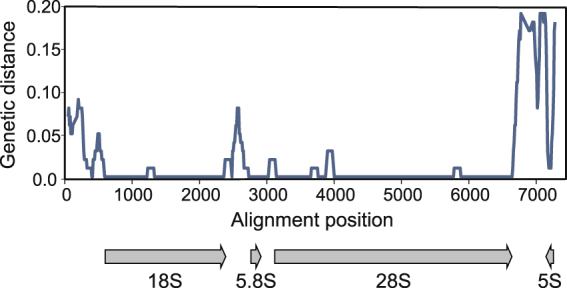
Nucleotide distance at rDNA locus. Sliding window approach (300 bp long window in 10 bp steps) was used to illustrate the conservation of nuclear rRNA genes between *C. glacialis* and *C. finmarchicus*. The locus is of similar size in both species but differs in the number of copies – there are approximately 1.6 times more copies of rDNA in genomic DNA of *C. glacialis.*

The most abundant repetitive element in both genomes is the rDNA gene cluster. The cluster apparently forms one or more arrays of tandem repeats, each containing the four rRNA genes (Fig. 7). The genetic distance between rRNA genes of the two studied *Calanus* species is remarkably low: there are no differences in *5.8 S*, one substitution in *18 S* and *5 S* and 34 substitutions in the longest *28 S* gene (still, more than 99% sequence similarity). The average coverage of the consensus sequence was very high: 8600 × for *C. glacialis* and 5300 × for *C. finmarchicus*, again indicating more redundant structure of the former genome.

## Discussion

The *C. glacialis* and *C. finmarchicus* mitogenomes, both longer than 27 kbp are the longest reported mitogenomes, not only within the superorder Gymnoplea: 17,910 bp in *C. hyperboreus* ^21^ and >20,460 bp in *C. sinicus* ^23^, but also among Copepoda in general, including the species from the superorder Podoplea, which are relatively short and range from 13,440 bp in *Caligus clemensi* ^27^ to 15,981 bp in *Paracyclopina nana* ^24^. Such extraordinary lengths of the *C. glacialis* and *C. finmarchicus* mitogenomes are caused by an unusual length of NCRs, a feature that was also observed in *C. sinicus* ^23^. The 20,674 bp long sequence of *C. glacialis* mitochondrial genome, announced recently^28^, is almost identical to contig 2 presented by us (>99% sequence similarity), extended at 3’ end with low complexity repeatable region of substantial length, therefore contrary to the description given by the authors, we believe that in fact this contig does not contain the complete set of mitochondrial genes (compare Fig. 1a).

The *C. glacialis* and *C. finmarchicus* mitogenomes are structurally similar to the mitochondrial genome of the North Pacific *Calanus sinicus* ^23^ and differ from the much more typical mitogenome of the deep-water, Arctic *Calanus hyperboreus* ^21^. However, given the relatively large differences in sequence and structure of *C. glacialis* and *C. finmarchicus* mitogenomes, it must be concluded that these two species are genetically distinct and have evolved independently for quite a long time. Thus, it seems unlikely that *C. glacialis* and *C. finmarchicus* can hybridize in the areas of sympatry^29^.

**Figure 8:**
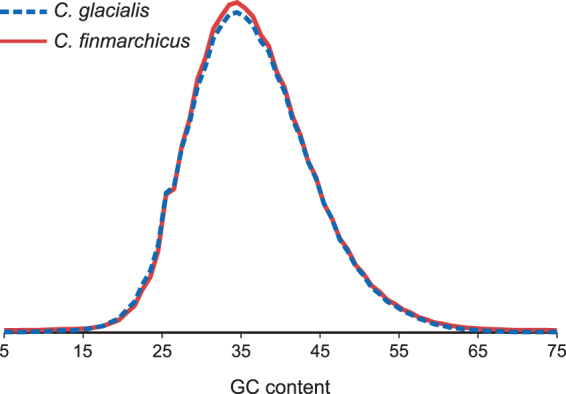
The relative distribution of GC (guanine-cytosine) content in genomic NGS reads. The two species do not differ in GC content, the two distributions are very similar (solid line represents *C. finmarchicus*). Note that the mitogenome GC content (42%) lies well within this range, which apparently precludes the separation of mitochondrial DNA by density gradients in these species.

The structural similarities in copepod mitogenomes parallel the genetic distances in this case (Fig. 3 and Fig. 4), reflecting the general evolutionary trend for the expansion of NCRs within mitochondrial genomes of *Calanus* species. An interesting consequence of this expansion is the unexpected property of their mitochondrial DNA: the AT-content of the sequenced part of the mitogenome appears to be similar to the average of the reported nuclear loci^30^, and lies within the range recorded for the sequenced nuclear DNA (Fig. 8), confirming that the separation of mitochondrial and nuclear DNA by density gradient centrifugation is impossible.

The structure of mitogenome in arthropods is considered relatively stable. The gene order close to the one found in the mitogenome of *Homarus americanus* is usually seen as a typical, ground pattern^21,27^. Copepods are exceptional arthropods because, with only a few complete mitogenomes published, they already present many deviations from the reference gene order^20,24,27,31–33^. However, the mitogenomes presented here are exceptional even among copepods. There is only one other known example of a congeneric mitogenomes in Copepoda: the two *Tigriopus* species from the order Hapacticoidea and these mitogenomes are much more stable. Even though they substantially differ at the sequence level (Fig. 4), their structure is similar. In fact, the only difference between these two genomes is the position of *trnW* gene, but a closer look at the actual alignment of the mitogenomes suggests that even this small difference is only caused by inconsistent annotation and not a real, structural difference. So, in the genus *Tigriopus*, in an evolutionary time long enough to accumulate substitutions in more than 25% of mitochondrial amino acid positions, there was no change in gene order. Contrary to that, the rate of rearrangements in the genus *Calanus* must be much greater, since despite the lower genetic distance (less than 20% aa p-distance, Fig. 4), there must have been at least four genomic rearrangements (Fig. 3), two involving large CDS and not just single *trn* genes as is usually the case^34^.

The expectation that the rearrangement and substitution rates are generally linked has been dismissed^35^, so in theory, this effect could be the result of slower-than-average amino acid substitution rate in *Calanus*. However, mitogenome rearrangements have also a noticeable effect on the local compositional biases, particularly if they involve NCRs. Negative GC skew at neutral sites has been used as an argument for reversal of the origin of replication in several species, including *Tigriopus* ^36,37^. In the mitogenome of *C. hyperboreus* the GC skew at neutral sites is also negative for all but the two shortest genes (Fig. 5). Similar conclusions have been reached when the data for all sites, not just the neutral ones, were used^21^. Still, the absolute skew values were smaller in *C. hyperboreus* than in *T. californicus*, suggesting less stable mutational pressure in *Calanus*. This is further confirmed by GC skew at neutral sites in the three C*alanus* species with multiple NCRs: *C. sinicus, C. finmarchicus* and *C. glacialis*. The absolute GC skews are even smaller in these species, and the sign of the skew frequently differs between genes, even the neighbouring ones (Fig. 5). This pattern suggests independent replication from different NCRs and/or frequent change of the mutational pressure associated with mitochondrial DNA replication. Therefore, the increased rearrangement rate and not the slower substitution rate is responsible for the observed pattern of polymorphisms in *Calanus*.

Our inability to close all the gaps by PCR is not totally unexpected, as similar difficulties were encountered by other teams sequencing *Calanus* mitogenomes^23^. Their results can be viewed as a limitation of PCR technology or as a proof of mitogenome fragmentation in *C. sinicus* ^23^. Similar considerations apply to the presented mitogenomes of *C. glacialis*, and even more so *C. finmarchicus*: the assembled contigs may actually represent separate mitochondrial “chromosomes”, existing without a constant physical linkage, even if they can be mapped on a circular mitogenome. The lack of consistent compositional biases could be used as an argument supporting such hypothesis. Fragmented, linear mitochromosomes of medusozoan cnidarians often evolve gene duplications^38,39^ and are terminated by inverted repeats. Nearly all the contigs assembled from *Calanus* mitogenomes are terminated with palindromic repeats. Moreover, in the case of *C. glacialis* mitogenome we report the existence of two *trnL2* genes, one per each contig. So there are some arguments supporting atypical structure of the reported mitogenomes, but they are not conclusive. Whatever the cause, these difficulties will not be solved without a breakthrough in sequencing technology allowing the reads to continue past difficult to amplify regions.

It has been argued that there is a relationship between body and genome size of keystone marine copepods^15,40^. Larger genomes were observed in cold waters species, a feature linked to slower growth and metabolic rates at lower temperatures^41,42^. The DNA content of a single nucleus is very high in the two studied species: 13 pg in *C. finmarchicus* and 24.2 pg in *C. glacialis* ^43^. According to^44^ this translates to 1.27 and 2.37 × 10^10^ bp per genome, respectively. Clearly, our sequencing effort (at 4.5 × 10^10^ bp) was not enough to achieve a more precise estimate of the genome size. However, if either of the two genomes was in fact highly polyploid, the achieved coverage could have been adequate to estimate the size of the haploid equivalent. This was not the case, hence no direct support for polyploidization. According to^45^ the two species differ in the concentration of rDNA in their genomes – the species with larger genome has also the higher concentration of rDNA, by approximately 17%. This suggests that the existing relationship between body and genome sizes in these species cannot be explained by simple polyploidization. Our data suggest even a greater disproportion in the concentration of rDNA between the two genomes (60% more rDNA in *C. glacialis*) and the expansion of certain classes of repeatable elements in this species.

In conclusion, large differences in the length and structure of *C. glacialis* and *C. finmarchicus* mitochondrial genomes indicate that the species are genetically distinct, so it is unlikely that they can hybridize. The molecular and evolutionary processes leading to the rapid structural evolution of both nuclear and mitochondrial genomes of these zooplankton species, key for the polar and sub-polar marine ecosystems, remain to be explained. We believe that the molecular resources presented here: the mitogenomic and rDNA sequences of the Arctic *C. glacialis* and its North Atlantic sibling *C. finmarchicus*, as well as the database of repetitive elements should facilitate the development of genetic markers suitable in pursuing this subject.

## Methods

### Sampling, DNA extraction and sequencing

*Calanus glacialis* was collected from the Hornsund fjord (station 77°00.54′N 16°28.3′E, western Svalbard Archipelago) in August 2012 (250 individuals) and 2013 (150 individuals) and *C. finmarchicus* from the Norwegian Sea (73°30.06′N 13°04.1′E) in June 2013 (700 individuals), during the routine scientific cruises onboard r/v Oceania. To facilitate species identification and handling, mainly the fifth copepodites were hand-picked and kept in 95% ethanol until use.

The strategy to sequence mitogenomes of the two species assumed that it will be possible to start with the sequences of mitochondrial transcripts, then design PCR primers and close the gaps by direct sequencing of PCR products^46,47^. To identify mitotranscripts we used RNA-Seq data of - SRA project accession number SRP053198 for *C. glacialis* ^30^ and the data of SRA project accession number SRP035889 for *C. finmarchicus* ^48^. Transcript assembly and identification followed the established pipeline^49^ and led to the identification of all CDS and rRNA mitotranscripts. Several PCR primers were designed and most gaps between the transcriptomic contigs were closed, as planned. In this round, we worked with 250 individuals of *C. glacialis* and 450 of *C. finmarchicus* from which we extracted DNA using a modified CTAB protocol^50^. However, technical difficulties associated with working with limited amount of starting material and frustrating failures in several seemingly well-designed PCRs precluded the realization of the original strategy. Moreover, since the overall genetic polymorphism in mitochondrial DNA of *C. glacialis* turned out to be very low^51^ we decided to continue the work with a heterogeneous sample of high quality DNA. Consequently, an attempt has been made to isolate pure mitochondrial DNA by density gradient centrifugation.

The procedure described by Lang and Burger^52^ was applied, isopyknotic caesium chloride gradient centrifugation was run for 16 h in an ultracentrifuge Beckman Coulter Optima L-90K with a vertical rotor. However, under long-wavelength UV we observed only a single DNA band, instead of expected separate mitochondrial and genomic DNA bands. Therefore, the procedure was repeated, starting with additional 150 individuals of *C. glacialis* and 250 of *C. finmarchicus*, with extended centrifugation time to 24 h. Again, only a single DNA band was obtained.

This highly purified DNA was used in NGS, in hope that the concentration of mitochondrial DNA would be high enough to allow a mitogenome assembly. To maximize our chances, one of the highest-throughput technologies available was used, the Illumina HiSeq X-Ten. Because of the high concentration and purity of DNA, the sequencing library was prepared using PCR-Free kit (Macrogen). The library has average insert size of 440 bp. The resulting set of approx. 3 × 10^8^, 150 bp long, paired end (PE) sequence reads, was used in all subsequent analyses.

### Gene identification and genome analysis

The preliminary identification of mitochondrial transcripts in RNA-Seq datasets^30^ was performed using estwise algorithm implemented in *wise2* software^53^, using hmm profiles of all mitochondrial protein coding sequences (CDS). Identified transcripts were then used as baits in MITObim^54^ and NOVOPlasty^55^ assemblers to retrieve the mitogenomes from the sets of NGS sequencing reads.

Gene annotations have been done semi-automatically. *De novo* prediction of all protein-coding genes was attempted using a set of algorithms implemented in CRITICA^56^, GLIMMER3^57^ and *wise2*. For prediction of RNA genes, ARWEN was used^58^. The available reasonable complete mitogenomes of the copepods from the same genus, *C. hyperboreus* ^21^ accession number: JX678968, *C. sinicus* ^23^ accession number: GU355641, were used to verify and fine-tune the annotations. The transcripts were mapped back onto the mitogenome contigs in CLC Genomics Workbench (version 9.5.4, QIAGENE) to verify consistency of gene annotations. Amino acid sequences for protein-coding genes were obtained using the invertebrate mitochondrial genetic code (NCBI translation table 5).

The obtained raw sequence reads are deposited in SRA database under the following accession numbers: SRR6063609 (*C. glacialis*) and SRR6065686 (*C. finmarchicus*), the assemblies are accessible through BioProject accessions: PRJNA411905 (*C. glacialis*) and PRJNA411907 (*C. finmarchicus*), the individual rDNA contigs are deposited in GenBank under accession numbers MF993123 and MF993124, mitochondrial contigs under MG001883 - MG001890.

There are several complete copepod mitogenomes in GenBank available for comparative analysis. However, they have a different organization and are also substantially distant from the mitogenome reported here, making meaningful comparison problematic. Therefore, the two congeneric sequences mentioned earlier were selected for a comparative analysis. The mitogenomic sequences of two harpacticoid copepod species from the genus *Tigriopus* were also used in some comparative analyses: *Tigriopus californicus* DQ917374^59^ and *Tigriopus* sp. AY959338^27^. All comparative analyses were performed following nucleotide alignment in MEGA7^60^, all nucleotide alignments were adjusted to correspond to amino acid alignments wherever possible, sites with alignment gaps were eliminated and bootstrap procedure with 1000 replicates was used to estimate standard errors.

For sequence manipulation such as extraction of genes and dotplot comparisons, EMBOSS suite was used^61^. Analysis of gene order data was performed using several heuristics implemented in CREx web server^62^. Analysis of k-mer frequency distributions were done in *Jellyfish* ^26^ and the assembly of repeatable DNA was done in *Tedna* ^63^, the provisional classification of the assembled elements was done by TEclass^64^. All mapping and manipulation of genomic reads were done in CLC Genomics Workbench.
